# Severe hypersomnia after unilateral infarction in the pulvinar nucleus– a case report

**DOI:** 10.1186/s12883-020-02018-2

**Published:** 2020-12-07

**Authors:** Peter Nørregaard Hansen, Thomas Krøigård, Nina Nguyen, Rune Vestergaard Frandsen, Poul Jørgen Jennum, Christoph P. Beier

**Affiliations:** 1 Department of Neurology, Odense University Hospital, Sdr. Boulevard 29, 5000 Odense, Denmark; 2 Department of Neurophysiology, Odense University Hospital, Odense, Denmark; 3 Department of Clinical Research, University of Southern Denmark, Odense, Denmark; 4 Department of Radiology, Odense University Hospital, Odense, Denmark; 5 Danish Center for Sleep Medicine, Department of Clinical Neurophysiology, Faculty of Health Sciences, University of Copenhagen, Rigshospitalet, Copenhagen, Denmark

**Keywords:** Thalamus, Pulvinar nucleus, Secondary hypersomnia, Stroke, Long sleep, Case report

## Abstract

**Background:**

Although a central role of the thalamus for sleep regulation is undisputed, the exact localization of the crucial structures within the thalamus remains controversial.

**Case presentation:**

Here we report a 35 year old woman with no prior comorbidities who developed severe and persistent hypersomnia with long sleep time after a small right-sided MRI-verified thalamic stroke affecting the dorsal part of the pulvinar and the dorsolateral boarders of the dorsomedial nuclei.

**Conclusion:**

The observed symptoms suggest a crucial role of posterior thalamus but not the midline parts of the thalamus in sleep-wake control.

**Supplementary Information:**

The online version contains supplementary material available at 10.1186/s12883-020-02018-2.

## Background

Despite the importance of sleep for humans, the anatomical structures involved in sleep regulation and the transition from sleep to wakefulness are complex and not fully understood. Two models for the transition from sleep to wakefulness have been proposed. The hypothalamus-brainstem mutual inhibition model, known as the flip-flop model, is based upon the interaction between the wake-promoting nuclei, the ascending reticular activating system, and the sleep-promoting nuclei, the ventrolateral and median preoptic nuclei [1]. The thalamo-cortical reverberation model suggests that variation of firing patterns in the thalamo-cortical network determine the vigilance state [2].

The role of the thalamus during sleep and the anatomic structures involved remains therefore elusive. The classical view of thalamo-cortical connection during sleep focused on primary sensory nuclei with reciprocal projections to primary sensory cortical areas [3]. However, the primary thalamus only discretely projects to small cortical regions and provides highly localized contribution to cortical sleep oscillation. Therefore, the thalamus may be unlikely to influence the global cortical activity during sleep [3]. The midline thalamus has been proposed as trigger of cortical up state [3], emphasized by receiving both projections from sleep-wake areas in the hypothalamus and brainstem, and efferent connections to widespread cortical areas [4, 5]. Most evidence in humans stems from stroke lesions. Only one study systematically assessed localization in a cohort of 12 patients with secondary hypersomnia, suggesting that the paramedian thalamus is part of the final common pathway required for maintenance of wakefulness [6]. Conversely, a study of thalamic activity during sleep suggested that the pulvinar thalamus is phase-advanced to the cortex at the onset of sleep [7] and possibly appears to be involved. Here, we report of a 35-year-old female patient with severe hypersomnia after unilateral posterolateral thalamic infarction that allows unique insights into the crucial structures involved in thalamic sleep-wake regulation.

## Case presentation

**Fig. 1:**
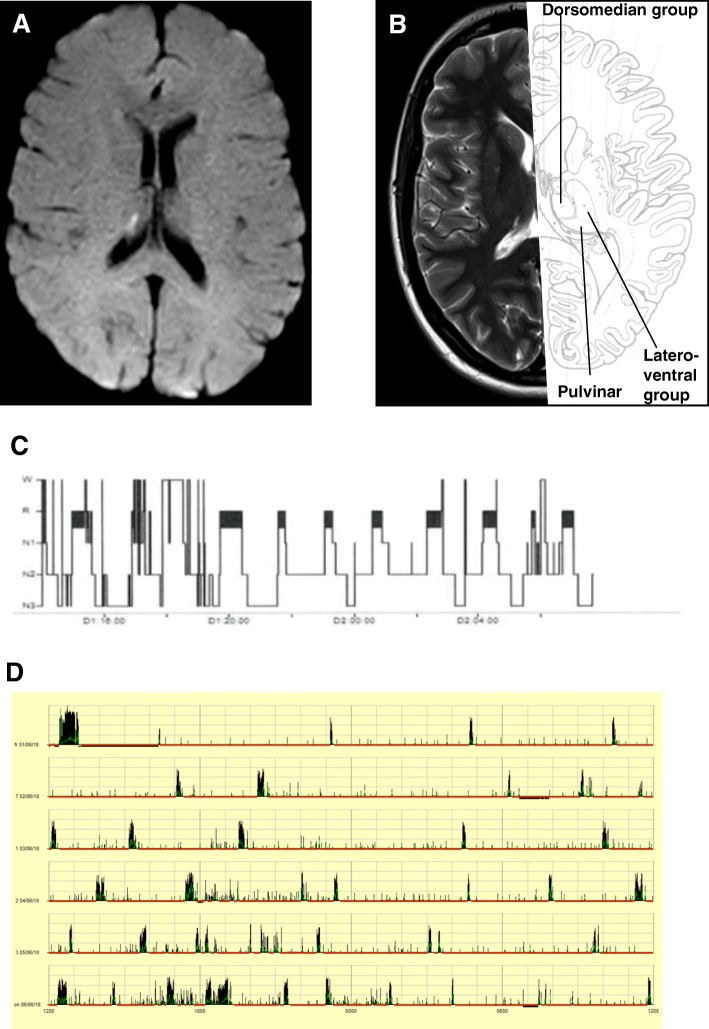
MRI, polysomography and actigraphy. **a** Acute diffusion weighted image showing the minor infarction in the right thalamus. **b** T1-weighted image combined with anatomical image [8] **c** Overview over sleep stages (hypnogram) from polysomnography performed May 2018. **d** Overview over actigraphy performed in June 2018

The patient gave written informed consent to this study, had no previous medical history (apart from migraine) and no history of substance abuse. In January 2016, she was acutely admitted to a local hospital with vertigo, nausea, headache and blurred vision. Vital signs and weight were normal (body mass index < 25). Neurological examination at admission revealed right sided hemihypesthesia. No ocular paresis or nystagmus was found. Acute 1.5 Tesla MRI showed an acute infarction in both cerebellar hemispheres and in the dorsal thalamus (dorsal part of the pulvinar and the dorsolateral boarders of the dorsomedial nuclei, Fig. 1a, b and Supplementary Figure 1). After admission, cognitive function evaluation using Montreal Cognitive Assessment was normal. Extensive search for etiology of the stroke remained inconclusive (incl. Echocardiography and screening for vasculitis). At discharge, the patient could move freely without assistance, only slightly impaired by vertigo. In addition, she developed severe daytime sleepiness that was first noticed by the relatives at discharge and did not improve since. Due to the lack of improvement of daytime sleepiness after more than 1 year, she was referred to our hospitals. A psychiatric evaluation was without signs of psychiatric disease, F-18 FDG PET/CT brain scan, sensory and motor evoked potentials, lumbar puncture (hypocretin concentration: 421 pg/mL) and blood test including corticotropin levels and thyroid status were normal. EEG 17 months later still showed frontal and bi-occipital slowing (Supplementary Figure 2). Three tesla MRI confirmed damage to the medial and posterior part of the right thalamus but no signs of infarction in the brain stem or new structural changes (Supplementary Figure 3). Polysomnography performed in May 2018 showed extensively prolonged total sleep time and abnormal short sleep latency (Fig. 1c) with almost continuous sleep with only few awakenings during day- and nighttime. Sleep structure and proportions of N1, N2, N3, and REM-sleep were normal apart from reduced frequency of sleep spindles. Multiple Sleep Latency Test (MSLT) showed continued sleep throughout the whole test period with sleep latencies < 30 s. Actigraphy verified the severely prolonged total sleep time (Fig. 1d). Treatment with modafinil (200 mg qd), methylphenidate (maximum of 120 mg qd) and pitolisant (maximum 36 mg qd) had no effect upon the symptoms. Current treatment with sodium oxybate (4.5 g × 2 at night) increased subjective wakefulness during the brief wake periods; however repeated PSG and MSLT performed before (November 2018) and during sodium oxybate treatment (May 2019) showed no significant changes in sleep structure or sleep time (Supplementary Figure 4A-B).

## Discussion and conclusion

The small size of the lesion in our patient with severe secondary hypersomnia offers the possibility to further localize the crucial thalamic structures involved in human in sleep regulation. In line with other clinical and experimental studies, we found a reduced proportion of sleep spindle and intermittent slowing in the EEG suggestive for a hypersomnia secondary to a thalamic lesion [6]. We found no signs of other structural changes in other parts of the brain, neither on the acute MRI nor on the 3 T MRI 2 years after the stroke supporting our interpretation of a secondary hypersomnia due to the thalamic lesion.

Although we assume a link between ischemic stroke and hypersomnia, we cannot exclude a common cause for both conditions. For instance, paucisymptomatic viral infections may be associated with hypersomnia but may also cause stroke due to transient coagulopathy [9, 10]. However, the patient received a work up for rare causes of stroke without signs of an undetected underlying disease explaining both symptoms. Thus, if our assumption of a causal link of MR-verified stroke and severe hypersomnia is correct, a very small, localized lesion to the pulvinar thalamic area with minor involvement of the dorsomedian nucleus was associated with extensively prolonged total sleep time and abnormally reduced sleep latency for more than 1 year after onset. This suggests that posterior thalamus may play a crucial role in sleep-wake control. Despite of the sometimes variable localization of the thalamic nuclei and the challenges of localizing the thalamic nuclei on MRI [11], we think that this case report contributes to the ongoing debates whether sleep a is focal or wide-spread process and whether sleep is centrally or locally controlled [12]. The fact that the patient years after the lesion still produces significant NREM sleep without circadian rhythmicity illustrates the crucial role of the thalamus for homeostatic sleep pressure but also challenges the two-process model [13]. The severity of the symptoms was surprising given a unilateral thalamic lesion, which differs from other case reports. The almost normal sleep architecture may also indicate that a failure of the awaking system [14] but the patient had neither clinical symptoms nor acute lesions in the brain stem, the basal forebrain or the hypothalamus on the first MRI. It is, however, difficult to exclude that the 1.5 T MRI have missed minor lesions in the brain stem. The severity of the symptoms suggests an increased individual susceptibility of this patient. Although 3 T MRI was otherwise structurally normal, it is tempting to speculate about an anatomical variant in this patient, e.g. a hypoplastic or functionally dysfunctional contralateral pulvinar nucleus. However, this individual susceptibility does not interfere with our main conclusion of our paper that the dorsal thalamus may be the crucial structure in humans for sleep regulation.

Two previous studies including 31 and 12 patients found that stroke lesions restricted to the paramedian thalamus were associated with hypersomnia. A combined total of 30 patients had unilateral thalamic lesions [6, 15]. Only Bassetti et al. [6] performed a detailed description of the lesions, and based on the patient data provided, the patients included had large and widespread lesions beyond the paramedian thalamus. We here add to this data by providing evidence that at small, well-defined lesion localized more lateral may also cause hypersomnia.

Thus, this patient’s history with a secondary hypersomnia likely caused by a small, well-defined stroke in the right pulvinar nucleus and minor damage of the dorsomedian nucleus of the thalamus may help to further localize the crucial thalamic structures in humans.

## Supplementary Information

**Additional file 1: Supplementary Figure 1.** Diffusion-weighted images of the 1.5 T MRI performed January 2016. Images showed infarction in the right cerebellar hemisphere and minor infarction sequelae in the right thalamus. **Supplementary Figure 2.** EEG from September 2018 showing intermittent bi-occipital slowing. **Supplementary Figure 3.** T2-weighted images of the 3 T MRI performed September 2018. Images showed minor infarction sequelae in the right cerebellar hemisphere and minor infarction sequelae in the right thalamus. No new lesions or lesion in the left thalamus were found. **Supplementary Figure 4.** Polysomnography before (**A**) and under (**B**) treatment with sodiumoxybat (4.5 g 2x at night).

## Data Availability

N.a.

## Abbreviations

MRI: Magnetic resonance imaging
FDG: Fluor-desoxy-glucose
PET: Position emission tomography
REM: Rapid eye movement
PSG: Polysomnography
MSLT: Multiple Sleep Latency Test
