# Supplementary material for: Severe hypersomnia after unilateral infarction in the pulvinar nucleus– a case report
Source: BMC Neurol. 2020 Dec 7;20:442. doi: 10.1186/s12883-020-02018-2 (PMC7720584; doi:10.1186/s12883-020-02018-2)

Supplementary Figure 1:

Diffusion-weighted images of the 1.5 tesla MRI performed January 2016. Images showed infarction in the right cerebellar hemisphere and minor infarction sequelae in the right thalamus.

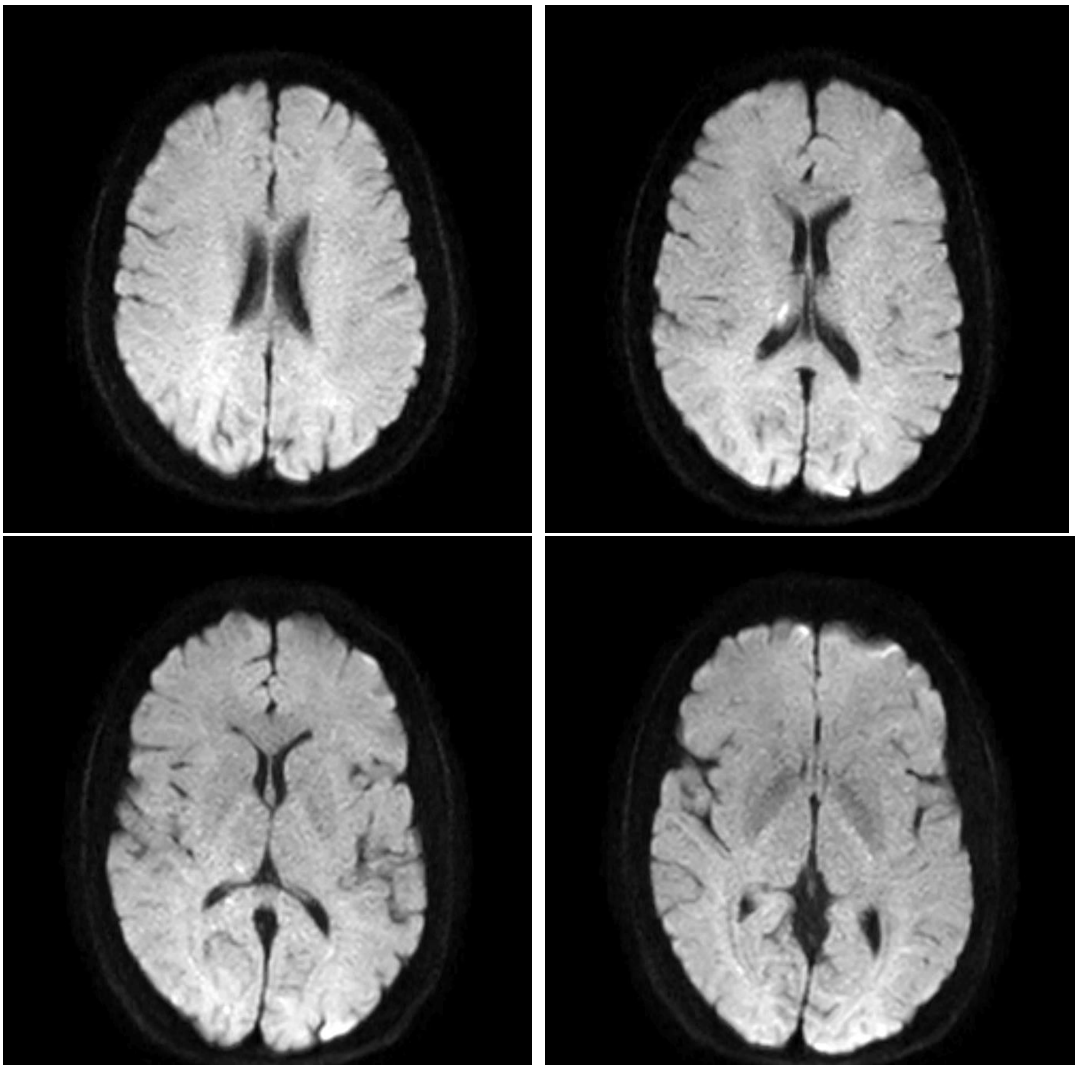

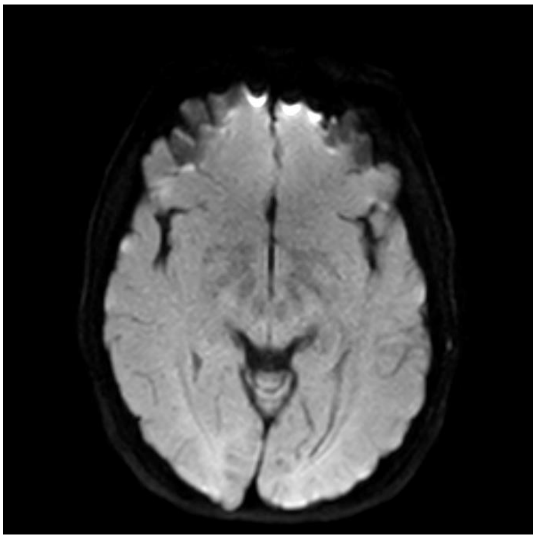
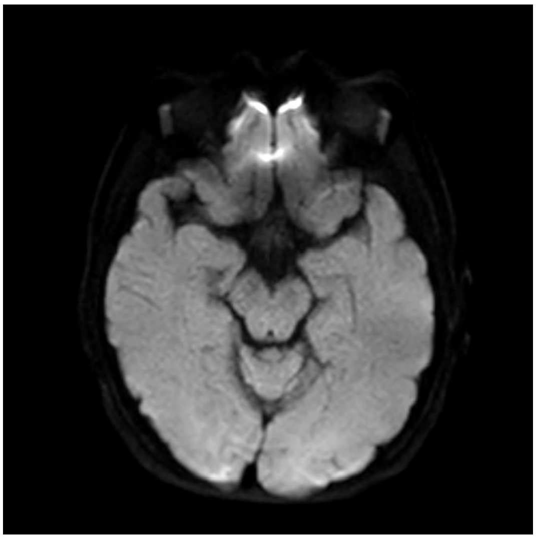
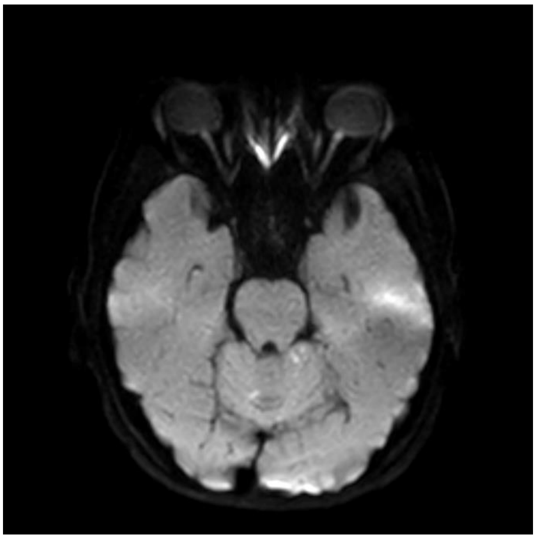
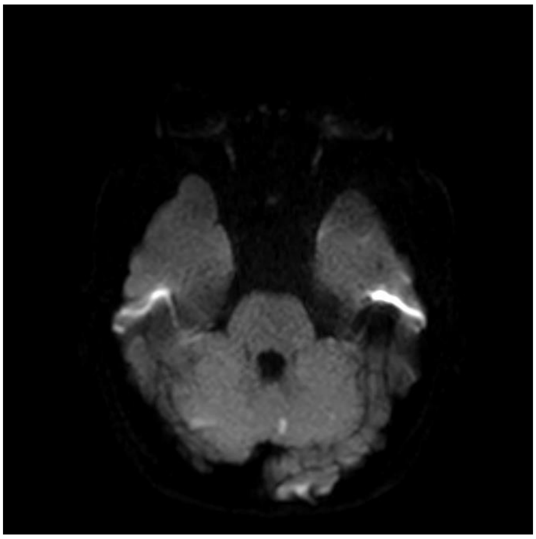
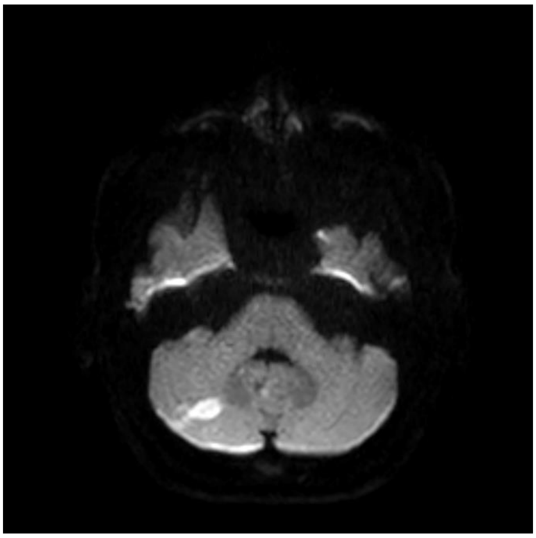
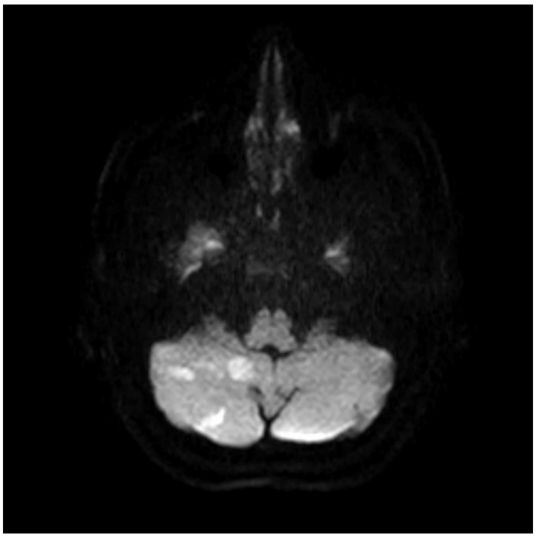

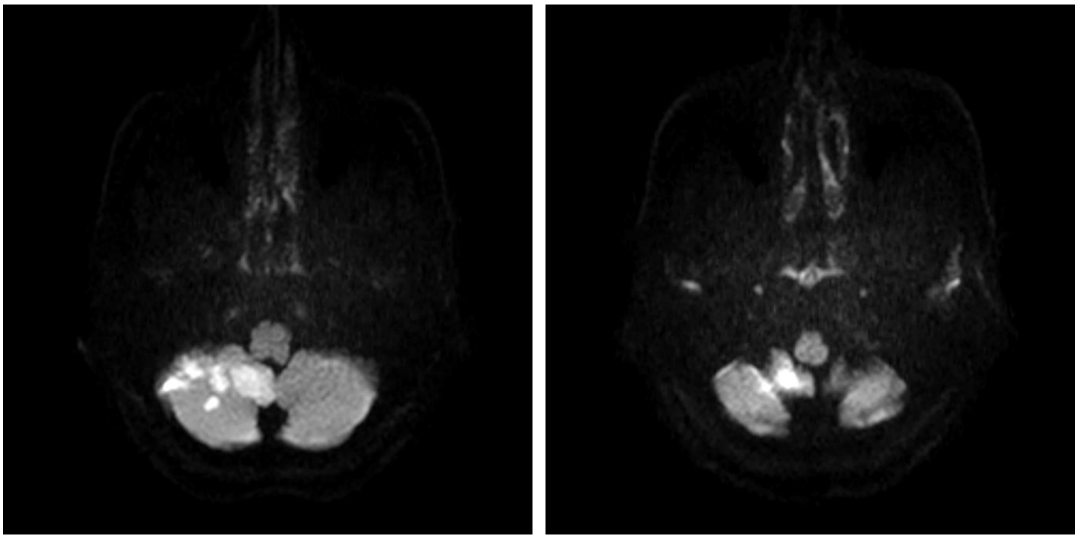

Supplementary Figure 2:

EEG from September 2018 showing intermittent bi-occipital slowing.

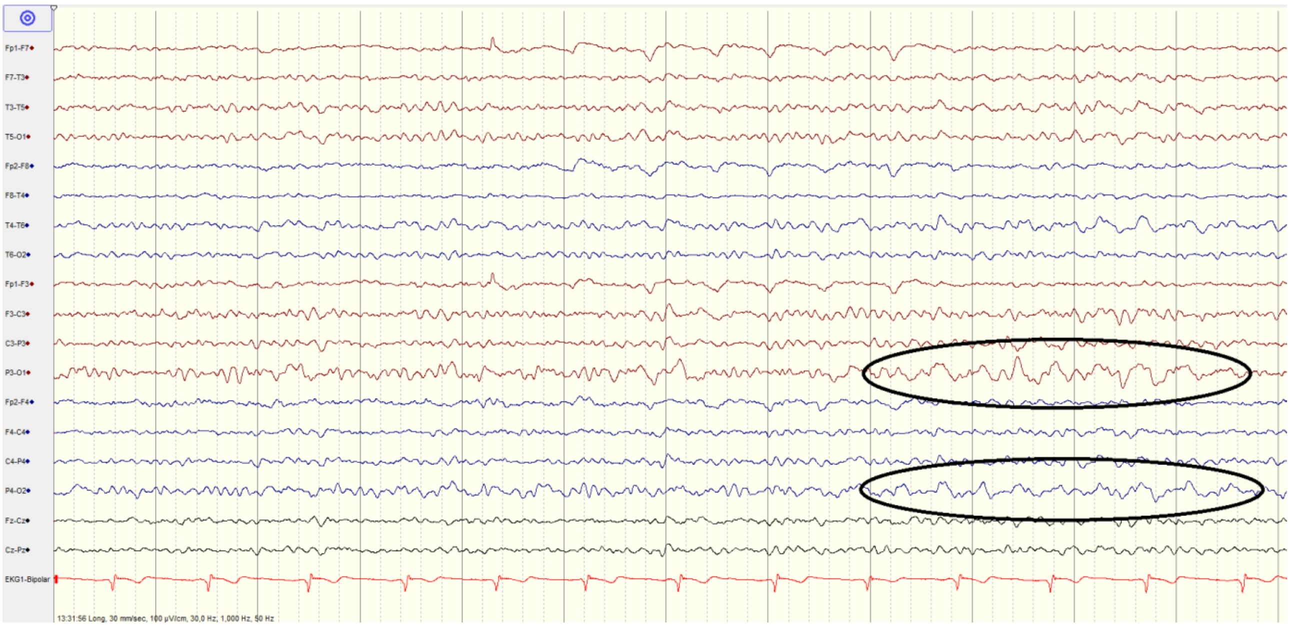

Supplementary Figure 3:

T2-weighted images of the 3 tesla MRI performed September 2018. Images showed minor infarction sequelae in the right cerebellar hemisphere and minor infarction sequelae in the right thalamus. No new lesions or lesion in the left thalamus were found.

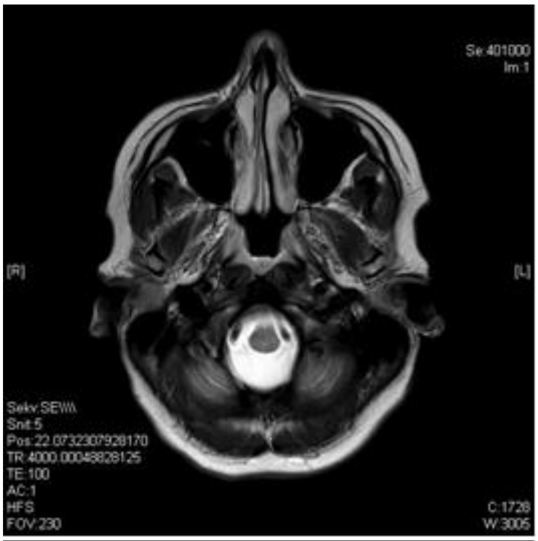
Se:401000
Im:1
[R]
[L]
Sekv:SE\\\
Snit:5
Pos:22.0732307928170
TR:4000.00048828125
TE:100
AC:1
HFS
FOV:230
C:1728
W:3005

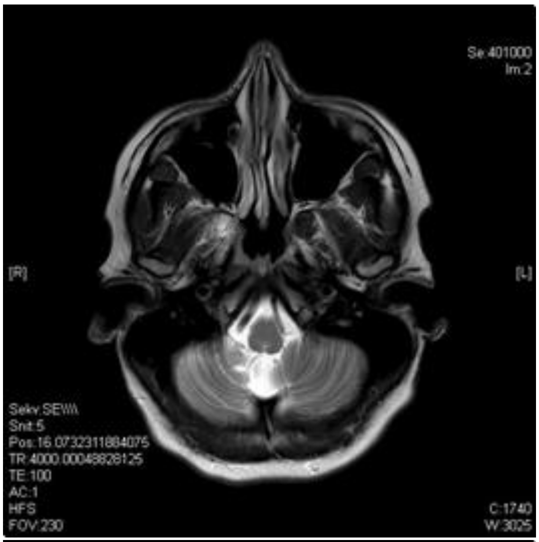
Se:401000
Im:2
[R]
[L]
Sekv:SE\\\
Snit:5
Pos:16.0732311884075
TR:4000.00048828125
TE:100
AC:1
HFS
FOV:230
C:1740
W:3025

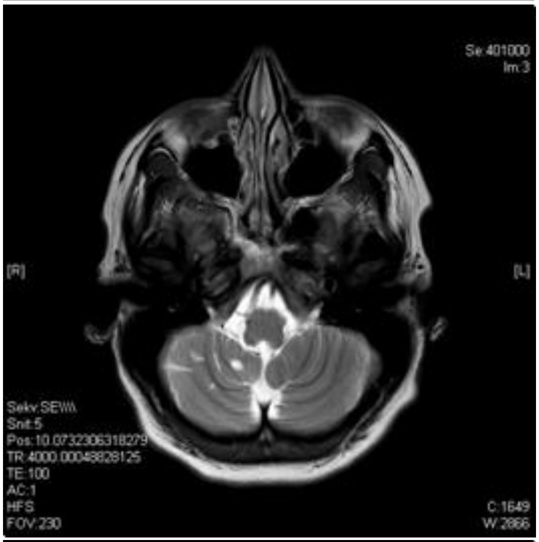
Se:401000
Im:3
[R]
[L]
Sekv:SE\\\
Snit:5
Pos:10.0732306318279
TR:4000.00048828125
TE:100
AC:1
HFS
FOV:230
C:1649
W:2866

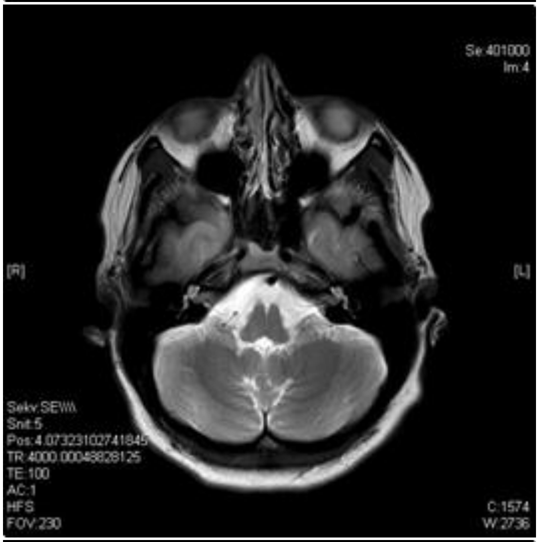
Se:401000
Im:4
[R]
[L]
Sekv:SE\\\
Snit:5
Pos:4.07323102741845
TR:4000.00048828125
TE:100
AC:1
HFS
FOV:230
C:1574
W:2736

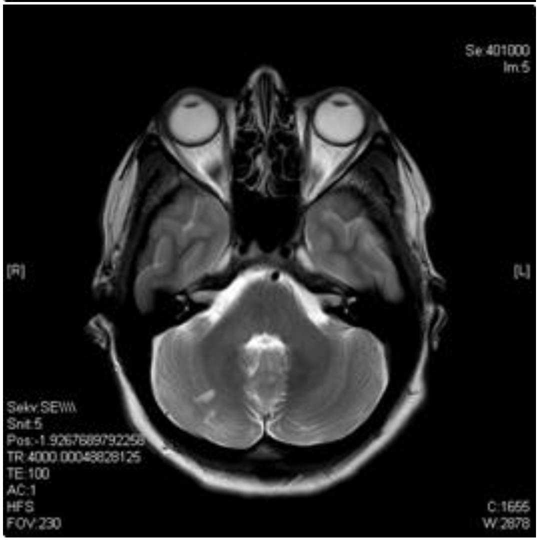
Se:401000
Im:5
[R]
[L]
Sekv:SE\\\
Snit:5
Pos:-1.9267689792258
TR:4000.00048828125
TE:100
AC:1
HFS
FOV:230
C:1655
W:2878

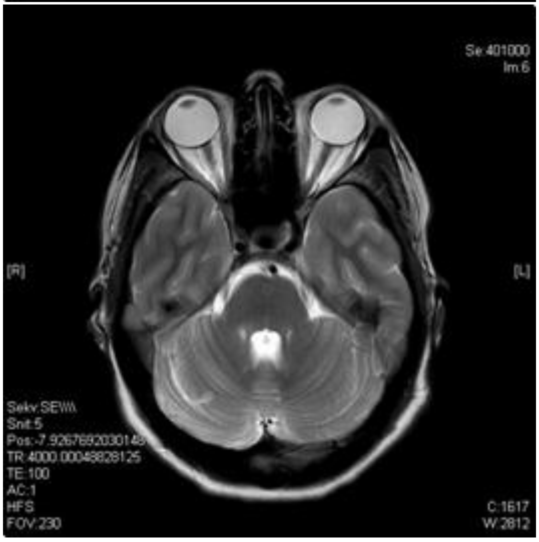
Se:401000
Im:6
[R]
[L]
Sekv:SE\\\
Snit:5
Pos:-7.9267692030148
TR:4000.00048828125
TE:100
AC:1
HFS
FOV:230
C:1617
W:2812

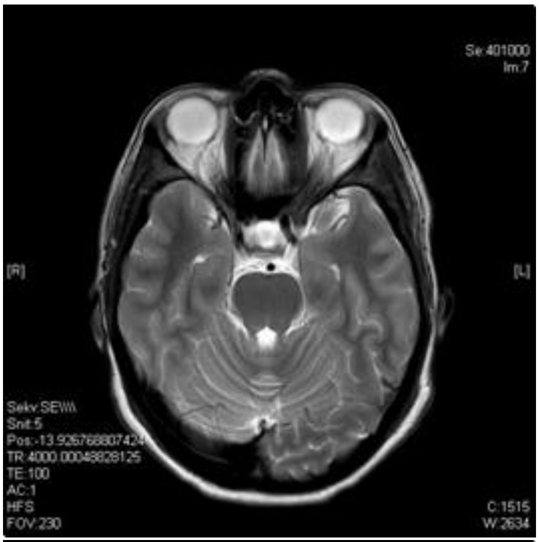
Se:401000
Im:7
[R]
[L]
Sekv:SE\\\\
Snit:5
Pos:-13.926768807424
TR:4000.00048828125
TE:100
AC:1
HFS
FOV:230
C:1515
W:2634

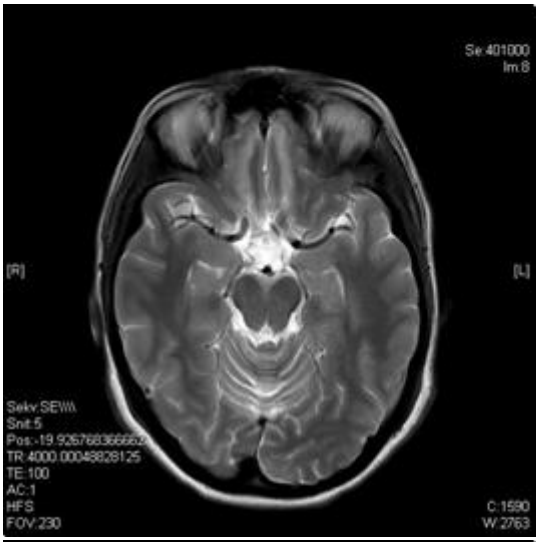
Se:401000
Im:8
[R]
[L]
Sekv:SE\\\\
Snit:5
Pos:-19.926768366662
TR:4000.00048828125
TE:100
AC:1
HFS
FOV:230
C:1590
W:2763

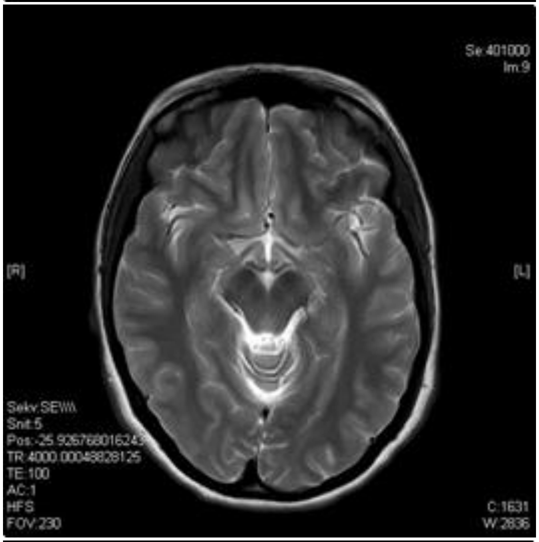
Se:401000
Im:9
[R]
[L]
Sekv:SE\\\\
Snit:5
Pos:-25.926768016243
TR:4000.00048828125
TE:100
AC:1
HFS
FOV:230
C:1631
W:2836

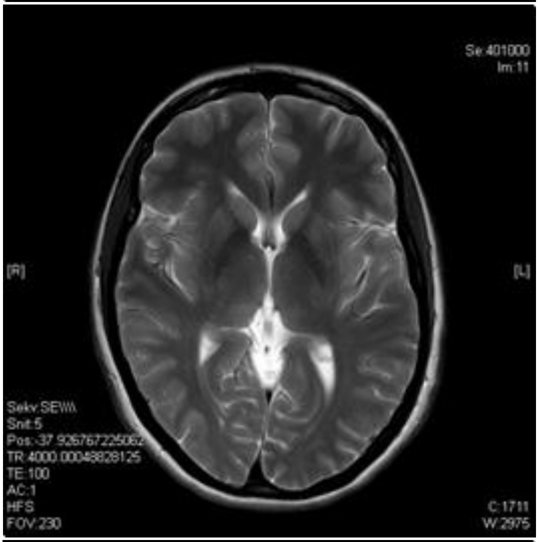
Se:401000
Im:11
[R]
[L]
Sekv:SE\\\\
Snit:5
Pos:-37.926767225062
TR:4000.00048828125
TE:100
AC:1
HFS
FOV:230
C:1711
W:2975

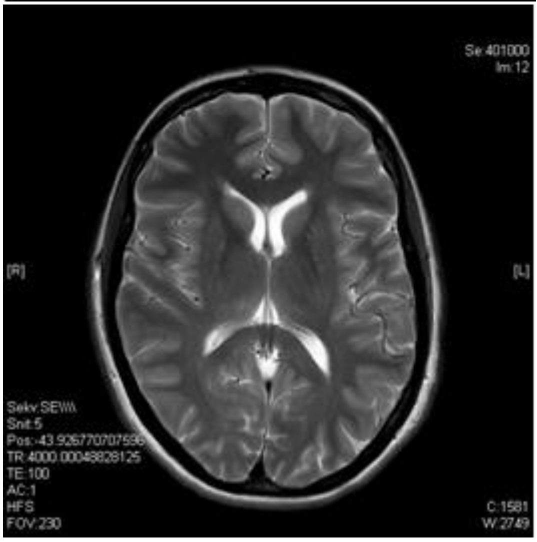
Se:401000
Im:12
[R]
[L]
Sekv:SE\\\\
Snit:5
Pos:-43.926770707596
TR:4000.00048828125
TE:100
AC:1
HFS
FOV:230
C:1581
W:2749

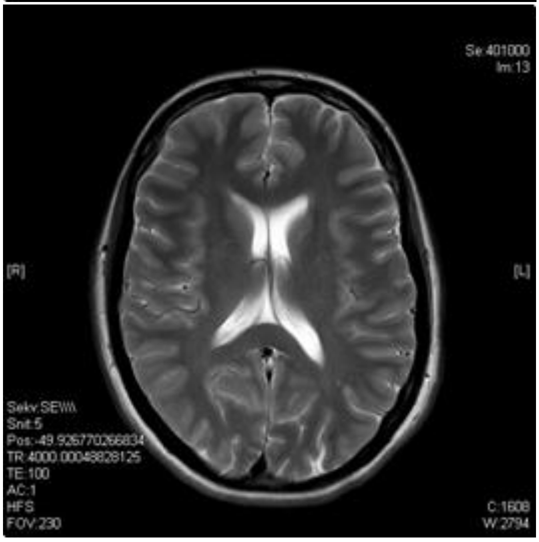
Se:401000
Im:13
[R]
[L]
Sekv:SE\\\\
Snit:5
Pos:-49.926770266834
TR:4000.00048828125
TE:100
AC:1
HFS
FOV:230
C:1608
W:2794

Supplementary Figure 4:

Polysomnography before (**A**) and under (**B**) treatment with sodiumoxybat (4.5 g 2x at night).

A

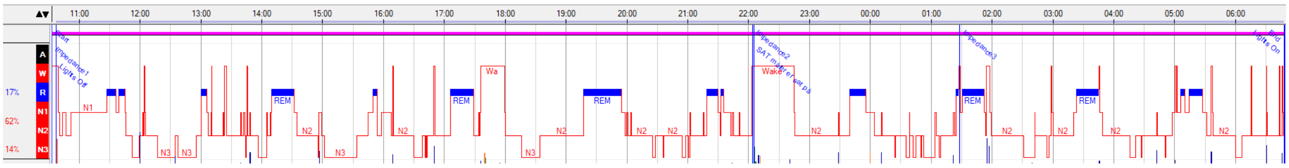

B

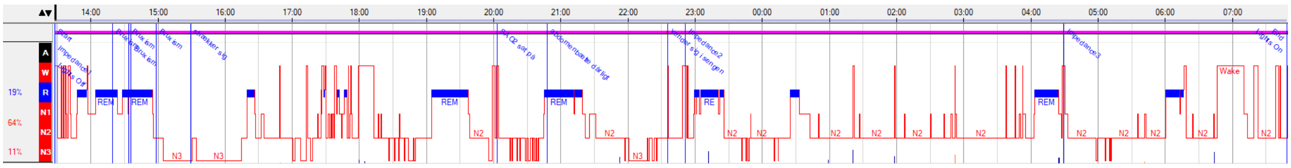

Supplement: Supplementary file 1 — Additional file 1: Supplementary Figure 1. Diffusion-weighted images of the 1.5 T MRI performed January 2016. Images showed infarction in the right cerebellar hemisphere and minor infarction sequelae in the right thalamus. Supplementary Figure 2. EEG from September 2018 showing intermittent bi-occipital slowing. Supplementary Figure 3. T2-weighted images of the 3 T MRI performed September 2018. Images showed minor infarction sequelae in the right cerebellar hemisphere and minor infarction sequelae in the right thalamus. No new lesions or lesion in the left thalamus were found. Supplementary Figure 4. Polysomnography before (A) and under (B) treatment with sodiumoxybat (4.5 g 2x at night). [file 12883_2020_2018_MOESM1_ESM.pdf]
